# Supplementary material for: Interpretable dimensionality reduction of single cell transcriptome data with deep generative models
Source: Nat Commun. 2018 May 21;9:2002. doi: 10.1038/s41467-018-04368-5 (PMC5962608; doi:10.1038/s41467-018-04368-5)
Supplement: Supplementary file 1 — Supplementary Information [file 41467_2018_4368_MOESM1_ESM.pdf]

Supplementary Figures for Ding *et al*:

Interpretable dimensionality reduction of single cell transcriptome data with deep generative models

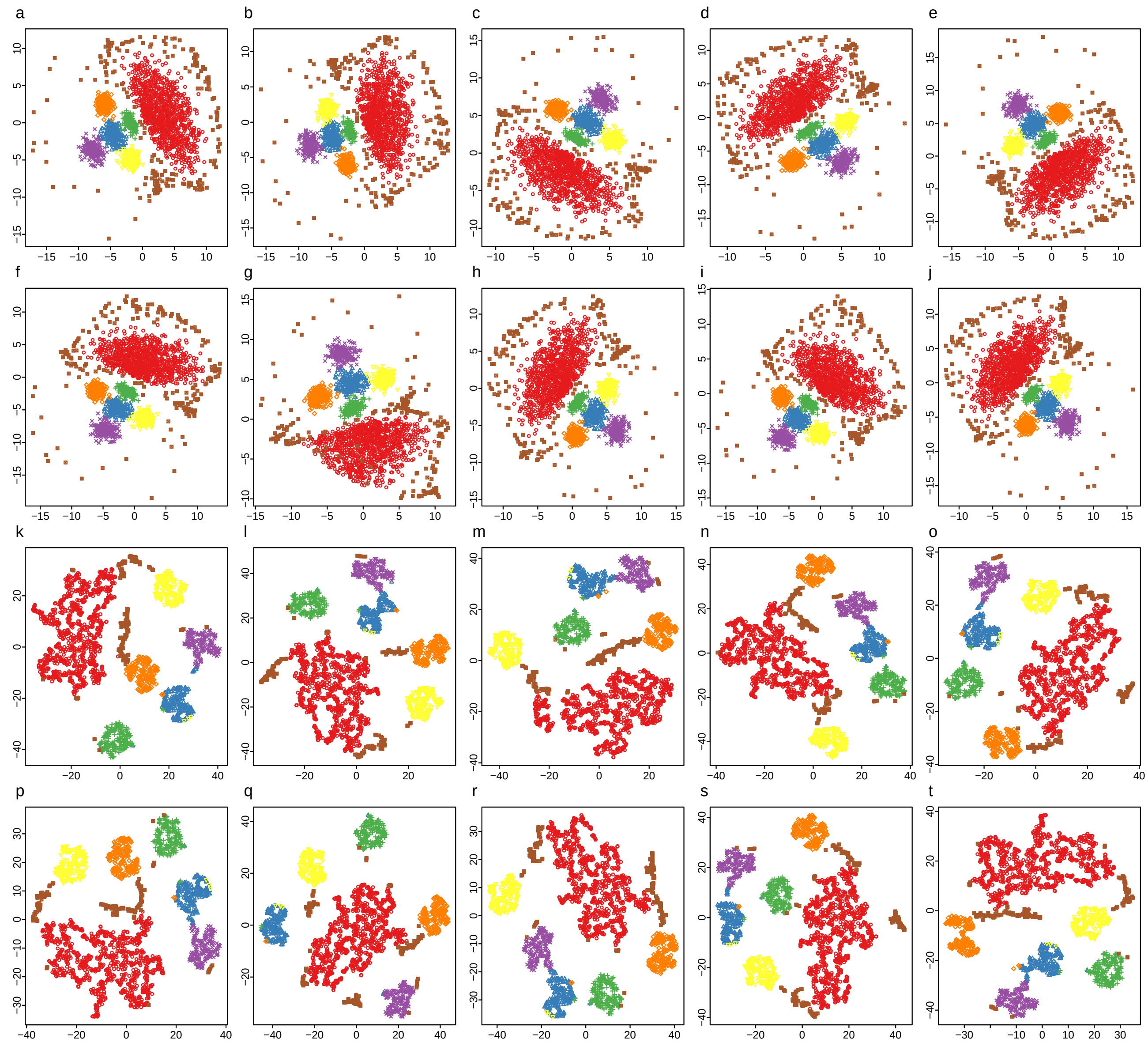

Supplementary Fig. 1: Repeated ten runs on the simulated nine-dimensional synthetic dataset (2,200 data points). (a-j) `scvis` results, and (k-t) t-SNE results. Here the color and symbol combinations encode clusters.

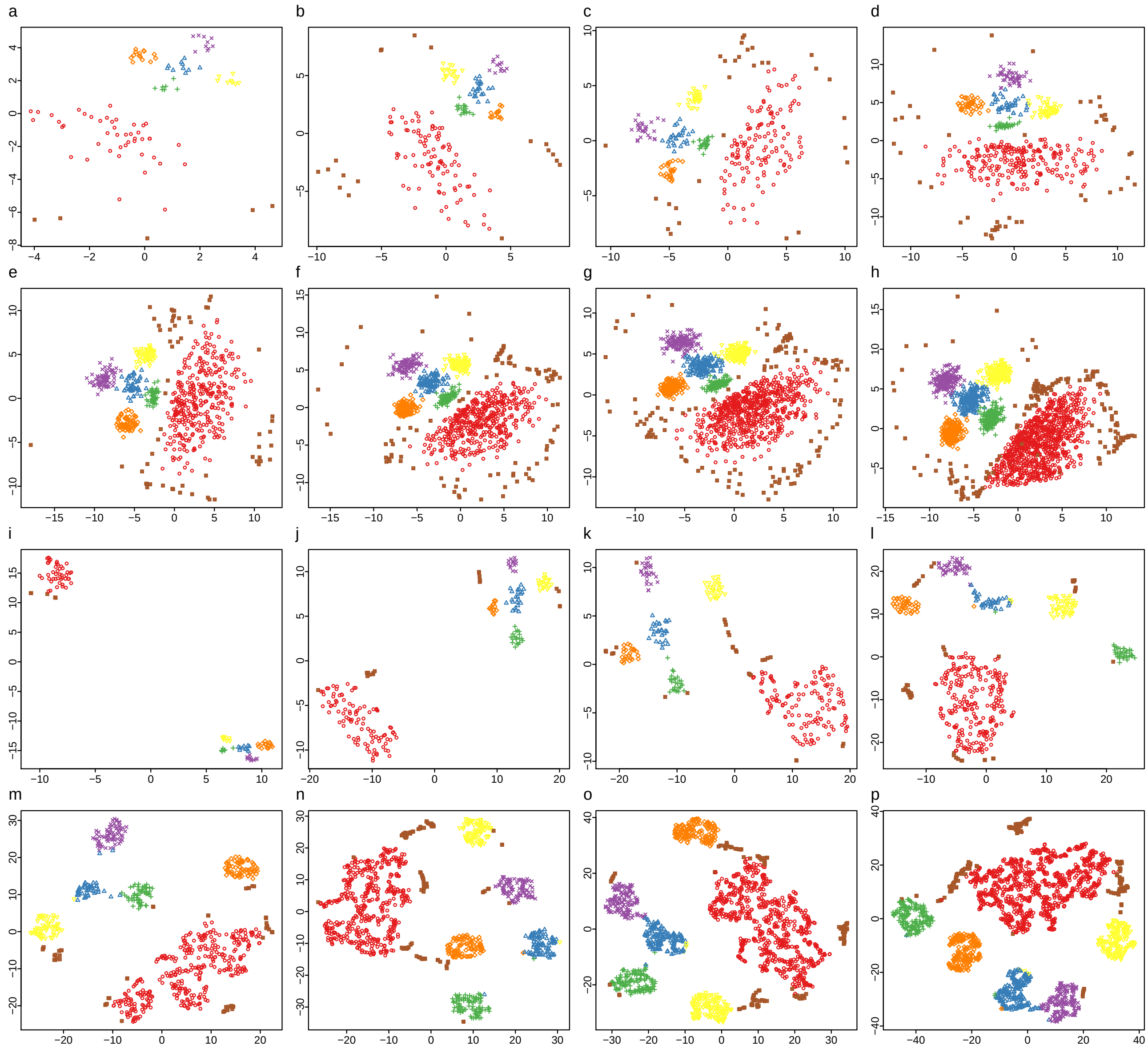

Supplementary Fig. 2: Sub-sampling analyses of the nine-dimensional synthetic dataset. We sub-sampled 100, 200, 300, 500, 700, 1000, 1,500, and 2,000 data points from the synthetic nine-dimensional dataset (with 2,200 data points). (a-h) `scvis` results, and (i-p) t-SNE results. Here the color and symbol combinations encode clusters.

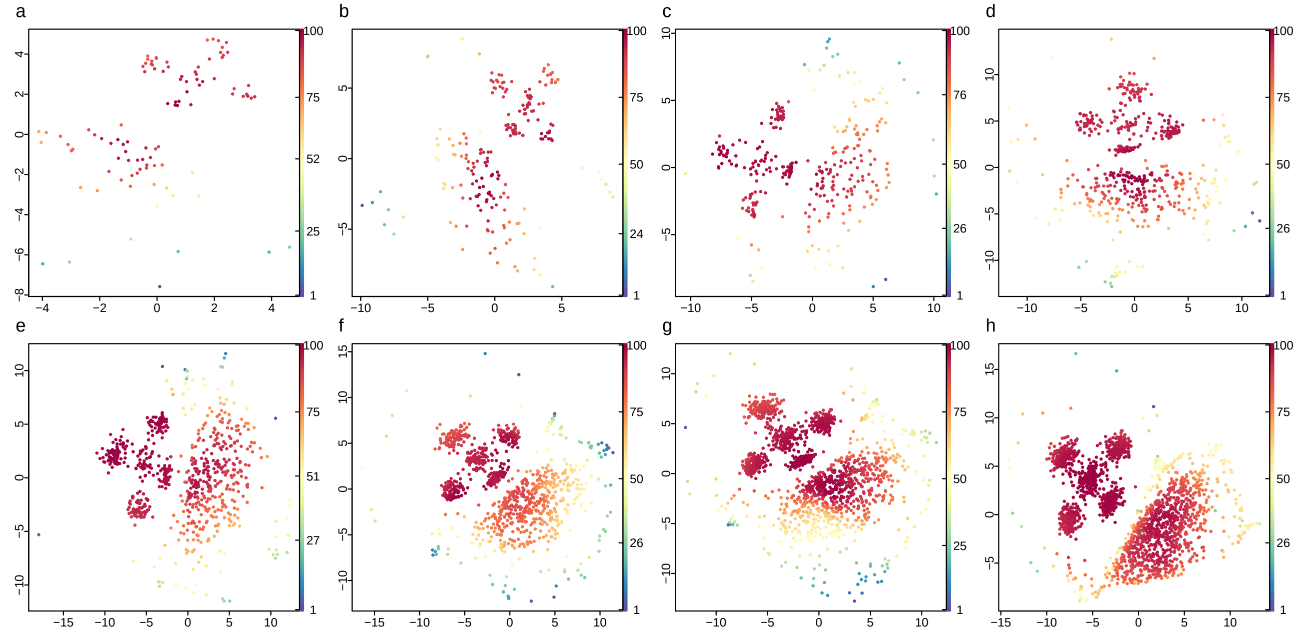

Supplementary Fig. 3: The estimated log-likelihoods from the sub-sampled data. a-h) `scvis` estimated log-likelihoods by training on sub-sampled 100, 200, 300, 500, 700, 1000, 1,500, and 2,000 data points from the synthetic nine-dimensional dataset with 2,200 data points. The colors encode the log-likelihoods.

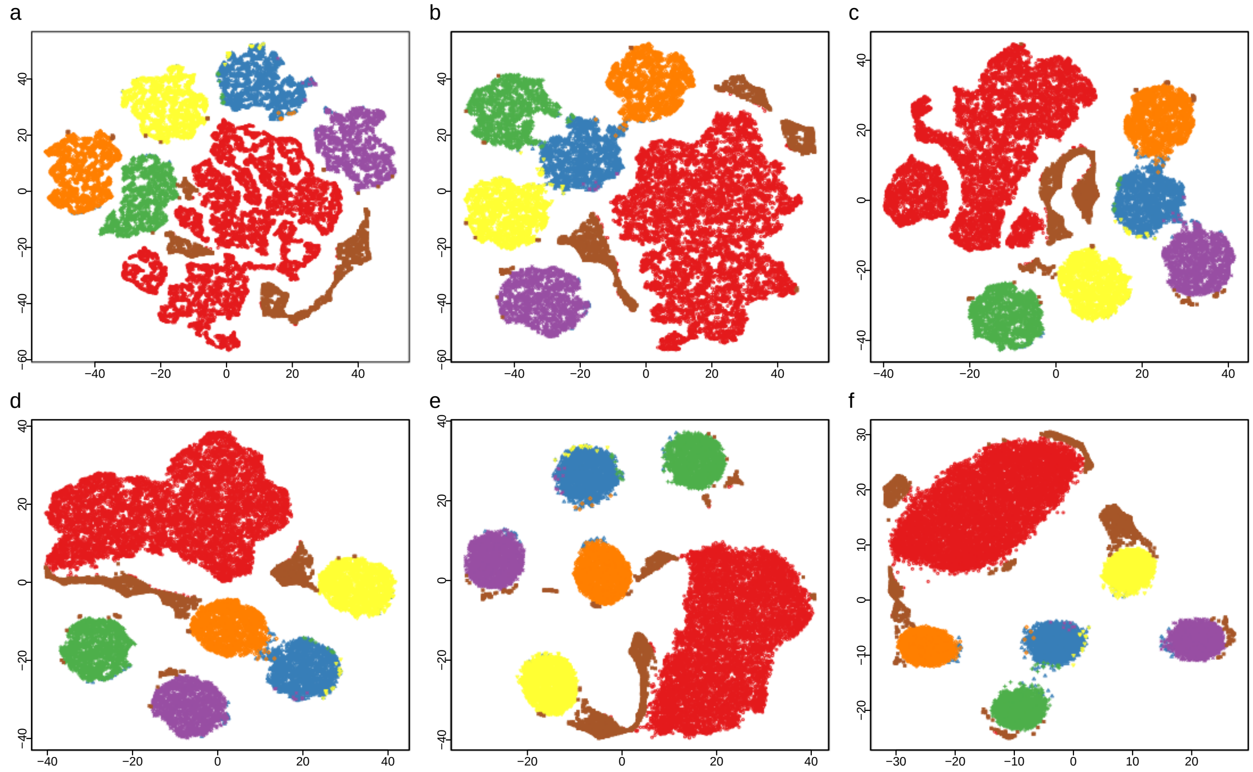

Supplementary Fig. 4: Performance of t-SNE on the 22,000 synthetic dataset with different perplexity parameters, a) 50, b) 100, c) 150, d) 200, e) 500, f) 1000. Here the color and symbol combinations encode clusters.

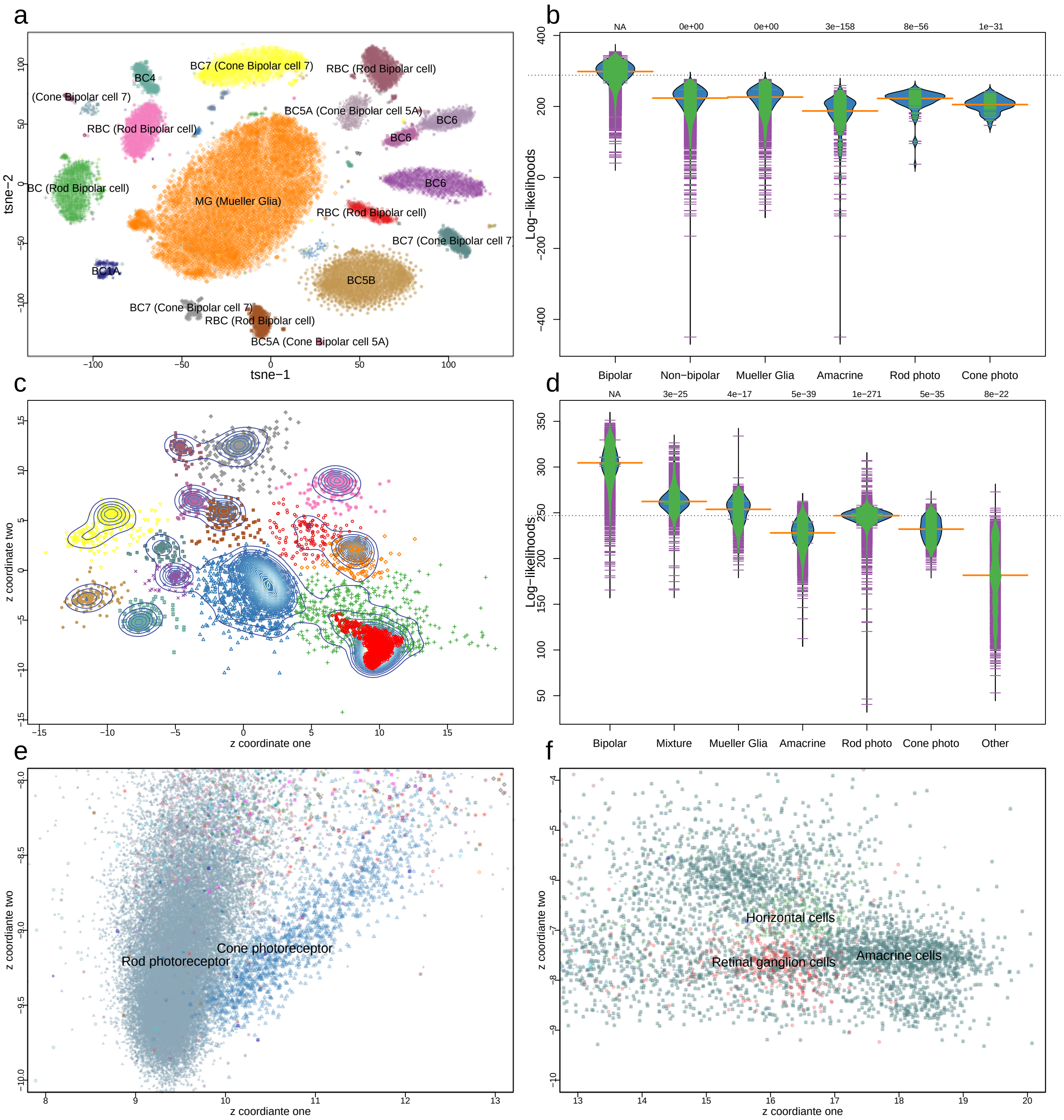

Supplementary Fig. 5: Learning a probabilistic mapping from the bipolar dataset. (a) The t-SNE results on the bipolar dataset, and the coordinates were from the original publication. (b) The log-likelihoods of points from the bipolar dataset were stratified by cell types, where the 'Non-bipolar' cells include Mueller Glia, Amacrine cells, Rod and Cone photoreceptors. The center values are the median, and the numbers at the top are the adjusted p-values (FDR, one-sided Mann-Whitney U-test, comparing the log-likelihoods of different cell types to those of bipolar cells). The orange center lines of the beanplots denote the mean. (c) `densitycut` clustering of the bipolar cells from the retina dataset, where the big mixture cell population is on the bottom right corner, and the high-density points (estimated from `densitycut` are labelled with red circles.) (d) The log-likelihoods of the cells from the retina dataset stratified by cell types, where the 'Other' cells were not observed in the training bipolar dataset. The center values are the median, and the numbers at the top are the adjusted p-values (FDR, one-sided Mann-Whitney U-test, comparing the log-likelihoods of different cell types to those of bipolar cells). The orange center lines of the beanplots denote the mean. (e) The Rod and Cone photoreceptors are mapped to the corresponding regions as in the training bipolar dataset. (f) The Amarcine cells, Horizontal cells, and Retina Ganglion cells are mapped to the same region.

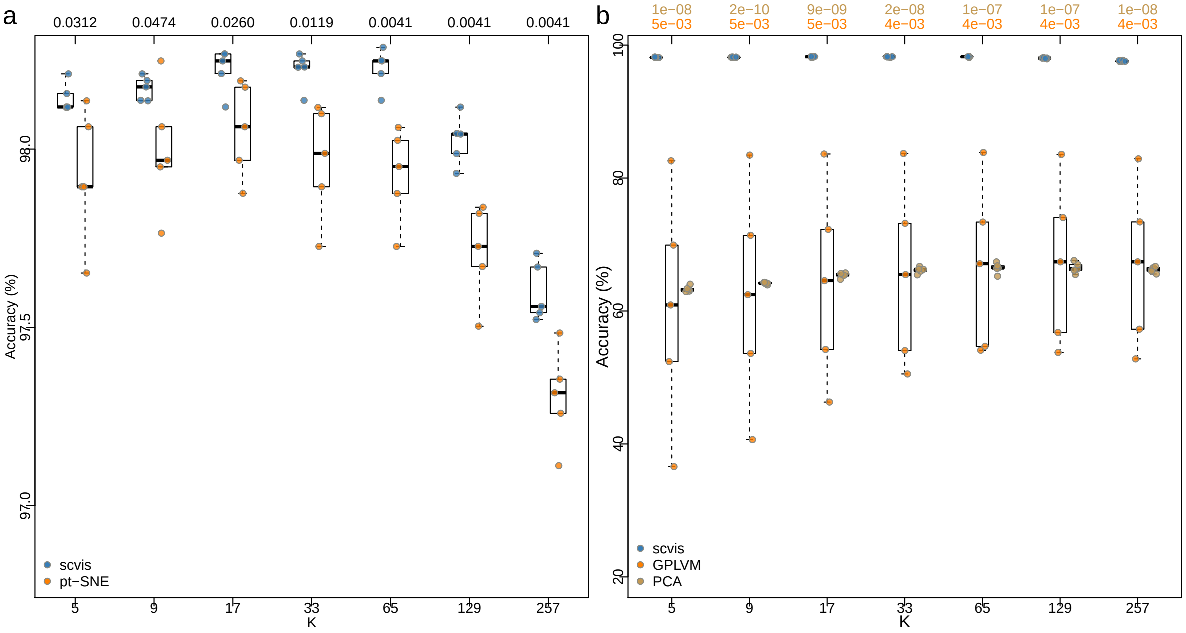

Supplementary Fig. 6: Five-fold cross validation benchmarking `scvis` against a) pt-SNE, b) GPLVM, and PCA in embedding the bipolar data. The numbers at the top are the FDR (one-sided Welch's t-test) comparing the Knn classification accuracies from `scvis` to those from (a) parametric t-SNE, and b) GPLVM and PCA. After learning different models from the training data, the held-out test data were mapped to two-dimensional spaces by the learned models. The Knn classifiers were trained on the two-dimensional coordinates of the training data and tested on the two-dimensional coordinates of the test data. Boxplots denote the medians and the interquartile ranges (IQR). The whiskers of a boxplot are the lowest datum still within 1.5 IQR of the lower quartile, and the highest datum still within 1.5 IQR of the upper quartile.

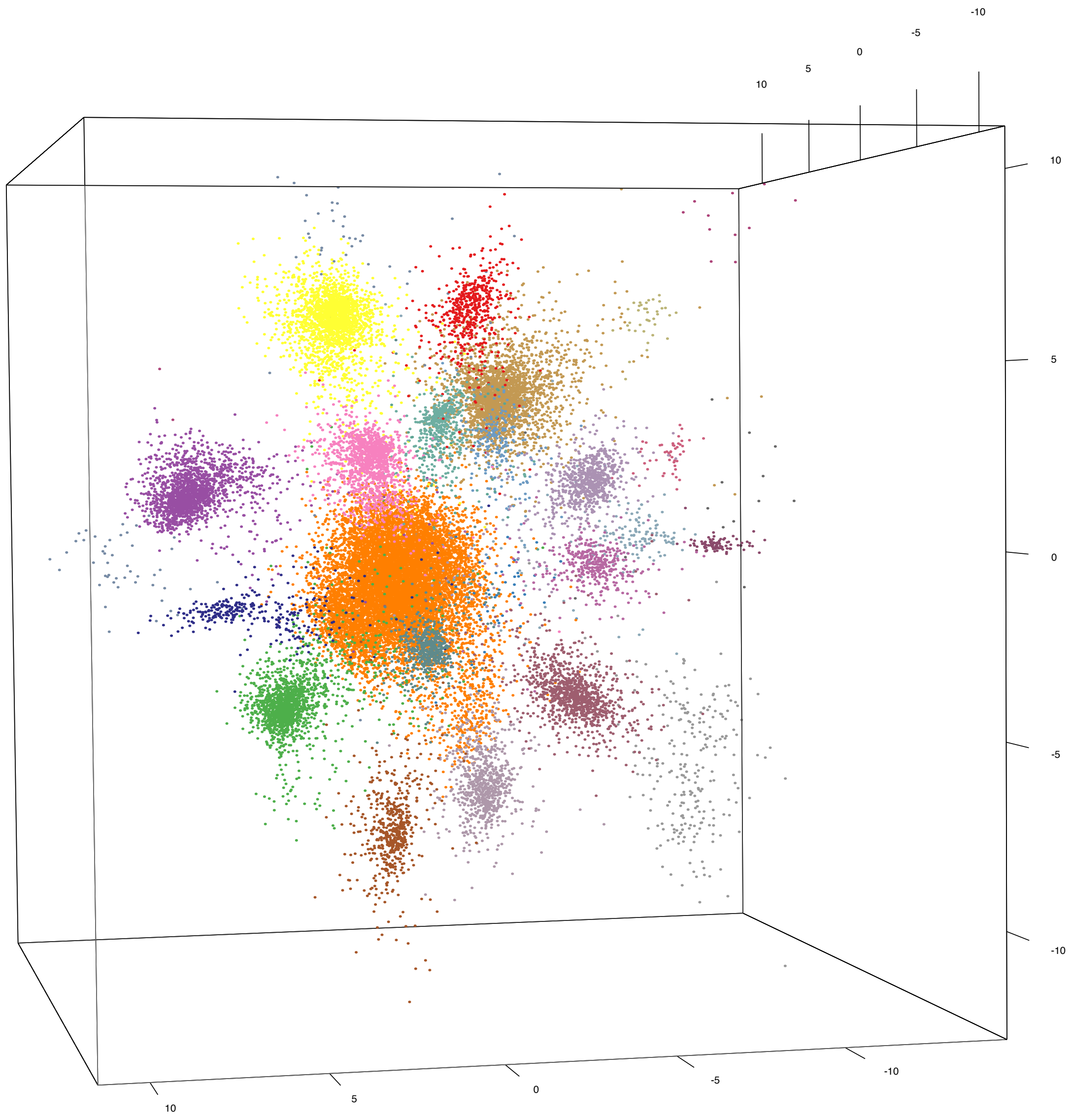

Supplementary Fig. 7: Projecting the bipolar data to a three-dimensional space. We obtained better average log-likelihood per data point, i.e., 255.1 versus 253.3 (from the last 100 iterations) by projecting the data to a three-dimensional space compared to projecting the data to a two-dimensional space. In addition, the average $\mathbb{KL}$ divergence is smaller (2.7 versus 4.1 from the last 100 iterations) by projecting the data to a three-dimensional space. Here the color and symbol combinations encode cells types.

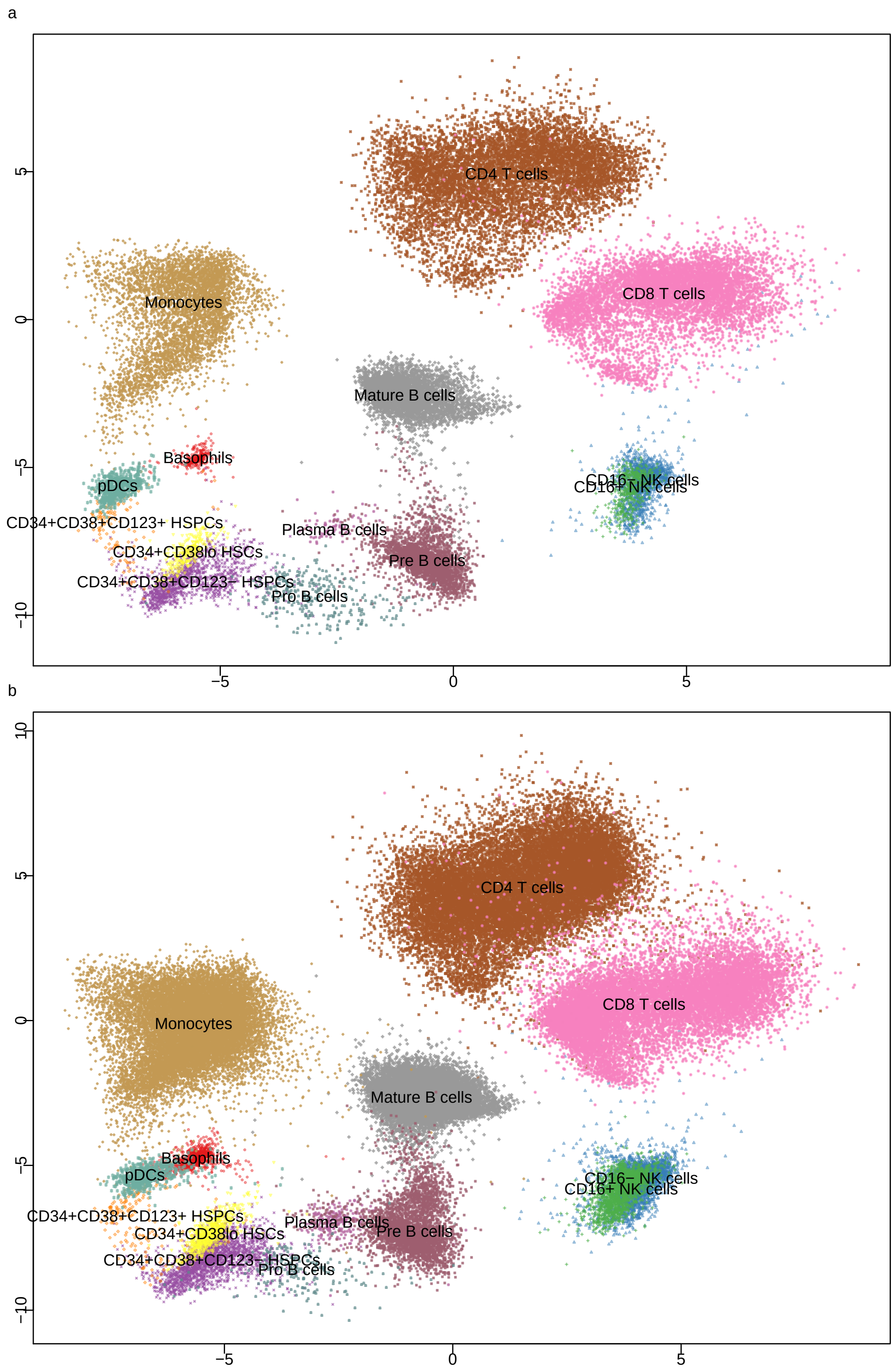

Supplementary Fig. 8: Results on the human single-cell mass cytometry data. (a) `scvis` results on the mass cytometry data from a healthy donor H2; (b) mapped the data from healthy donor H1 using the mapping function learned from H2. Here the color and symbol combinations encode cells types (cluster labels from the original publication).

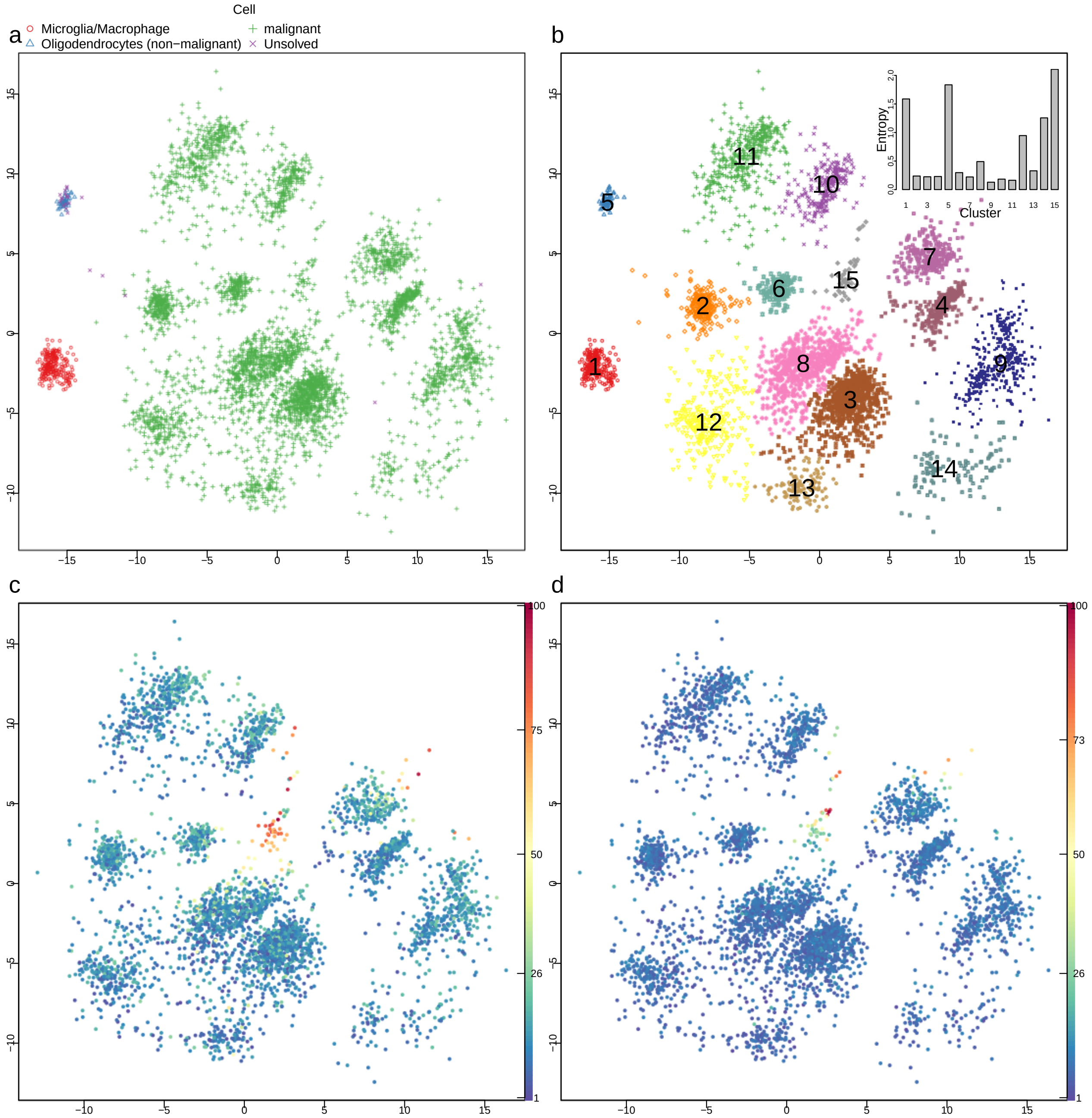

Supplementary Fig. 9: Analyzing intra-tumour heterogeneity. (a) Most cells in the oligodendroglioma dataset are malignant cells, and the non-malignant cells form two clusters are on the left. (b) `densitycut` analysis of the oligodendroglioma dataset, clustering one, five, and 15 have the highest entropies (based on the patients of origins of cells) (c) clustering 15 cells have either or both high G1/S scores and (d) G2/M scores (orange to red color represent high values).

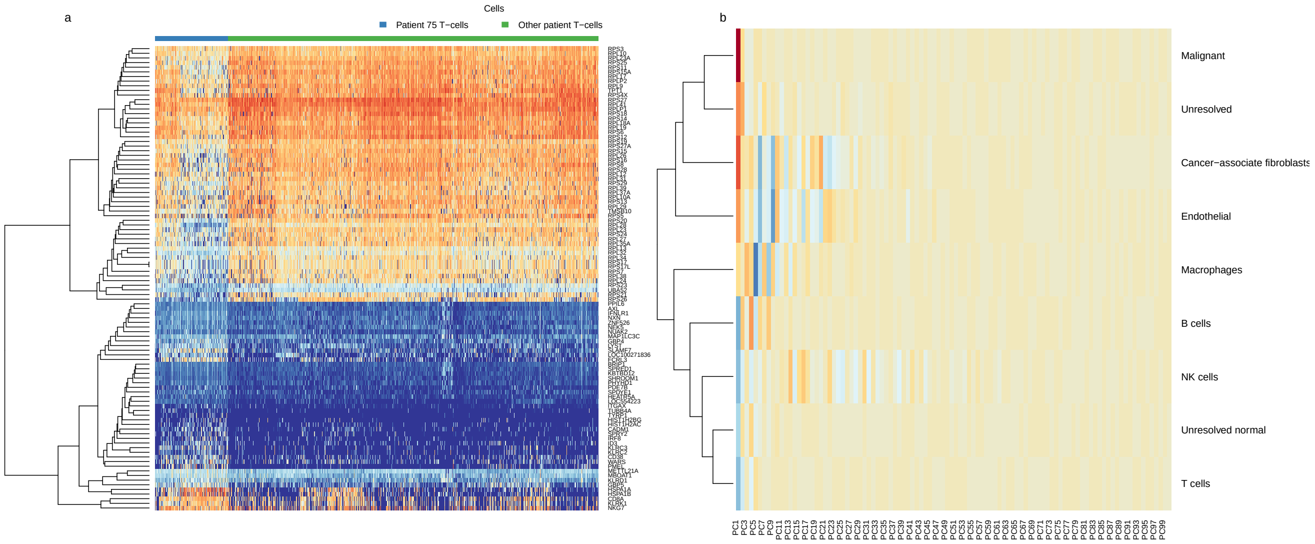

Supplementary Fig. 10: Analyzing intra-tumour heterogeneity. (a) The top 100 differentially expressed genes between patient 75 T cells and other patient T cells. (b) Hierarchical clustering of the average principal component values of different types of cells. Here red color represents high values and blue color represents low values.

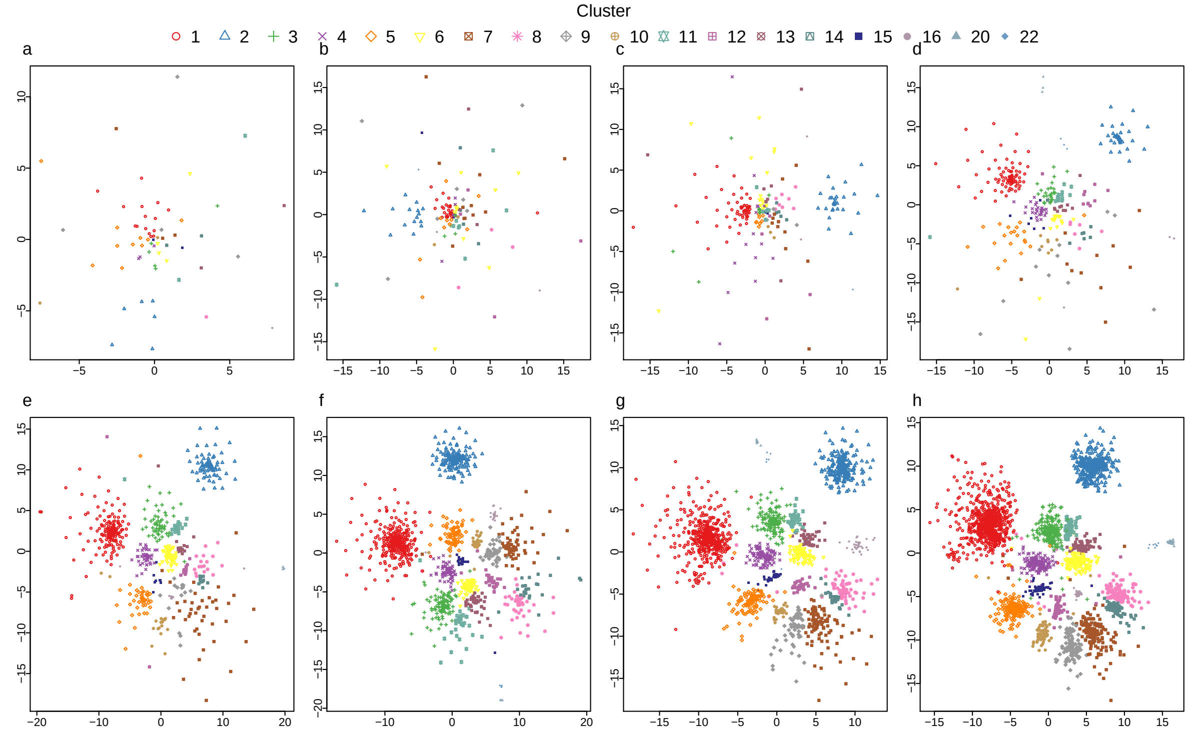

Supplementary Fig. 11: `scvis` results on the subsampled training bipolar dataset (with a random seed of 1) from batch six (6,221 cells in total after removing cell doublets and contaminants). Subsamapled a) 62 cells, b) 124 cells, c) 187 cells, d) 311 cells, e) 622 cells, f) 1,244 cells, g) 1,866 cells, and h) 3,110 cells. Here the color and symbol combinations encode cells types (clusters).

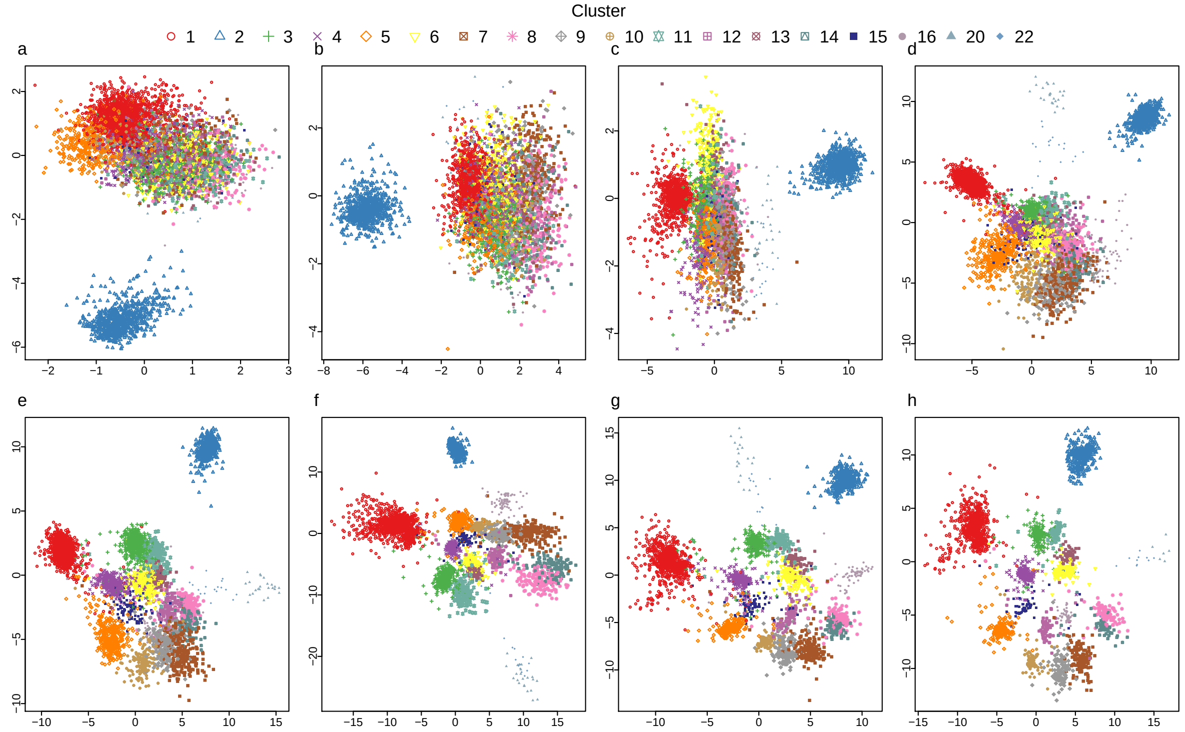

Supplementary Fig. 12: `scvis` results on the remaining test bipolar datasets from batch six (by using the mapping functions from the training data), a) 6,159 cells, b) 6,097 cells, c) 6,034 cells, d) 5,910 cells, e) 5,599 cells, f) 4,977 cells, g) 4,355 cells, and h) 3,111 cells. Here the color and symbol combinations encode cells types (clusters).

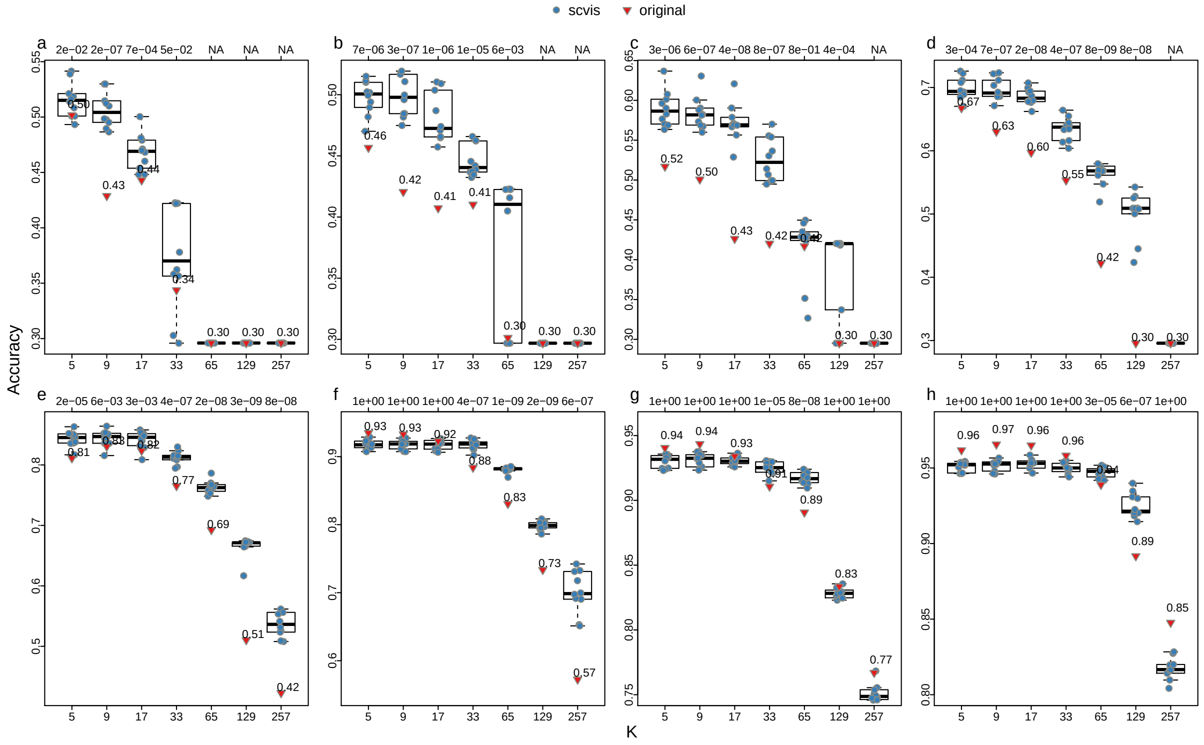

Supplementary Fig. 13: K-nearest neighbor classification accuracies on the test data trained on the `scvis` two-dimensional data (`scvis`) and the 100-dimensional principal components (original). The adjusted p-values (FDR, one-sided one-sample t-test) are shown at the top of each figure. Models trained on the subsampled data with a) 62 cells, b) 124 cells, c) 187 cells, d) 311 cells, e) 622 cells, f) 1,244 cells, g) 1,866 cells, and h) 3,110 cells. Boxplots denote the medians and the interquartile ranges (IQR). The whiskers of a boxplot are the lowest datum still within 1.5 IQR of the lower quartile, and the highest datum still within 1.5 IQR of the upper quartile.

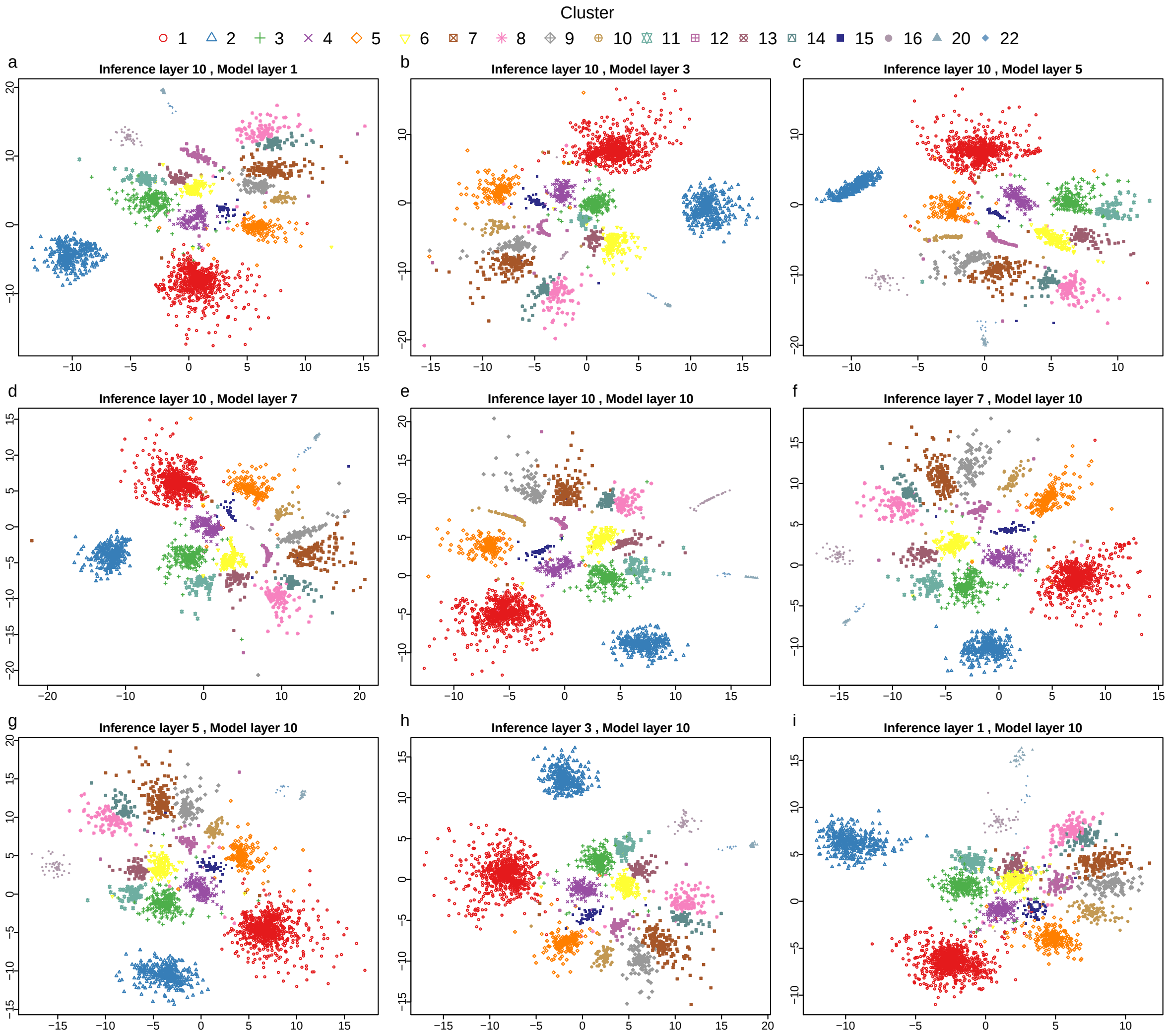

Supplementary Fig. 14: The influence of the number of layers in the neural networks on `scvis` results. We used the subsampled 3,110 training bipolar dataset from batch six. a-e) the inference networks have 10 layers, each layer has 128 units, and the model network has 1, 3, 5, 7, 10 layers, respectively. f-i) the model networks have 10 layers, each layer has 128 units, and the influence network has 7, 5, 3, 1 layers, respectively. Here the color and symbol combinations encode cells types (clusters).

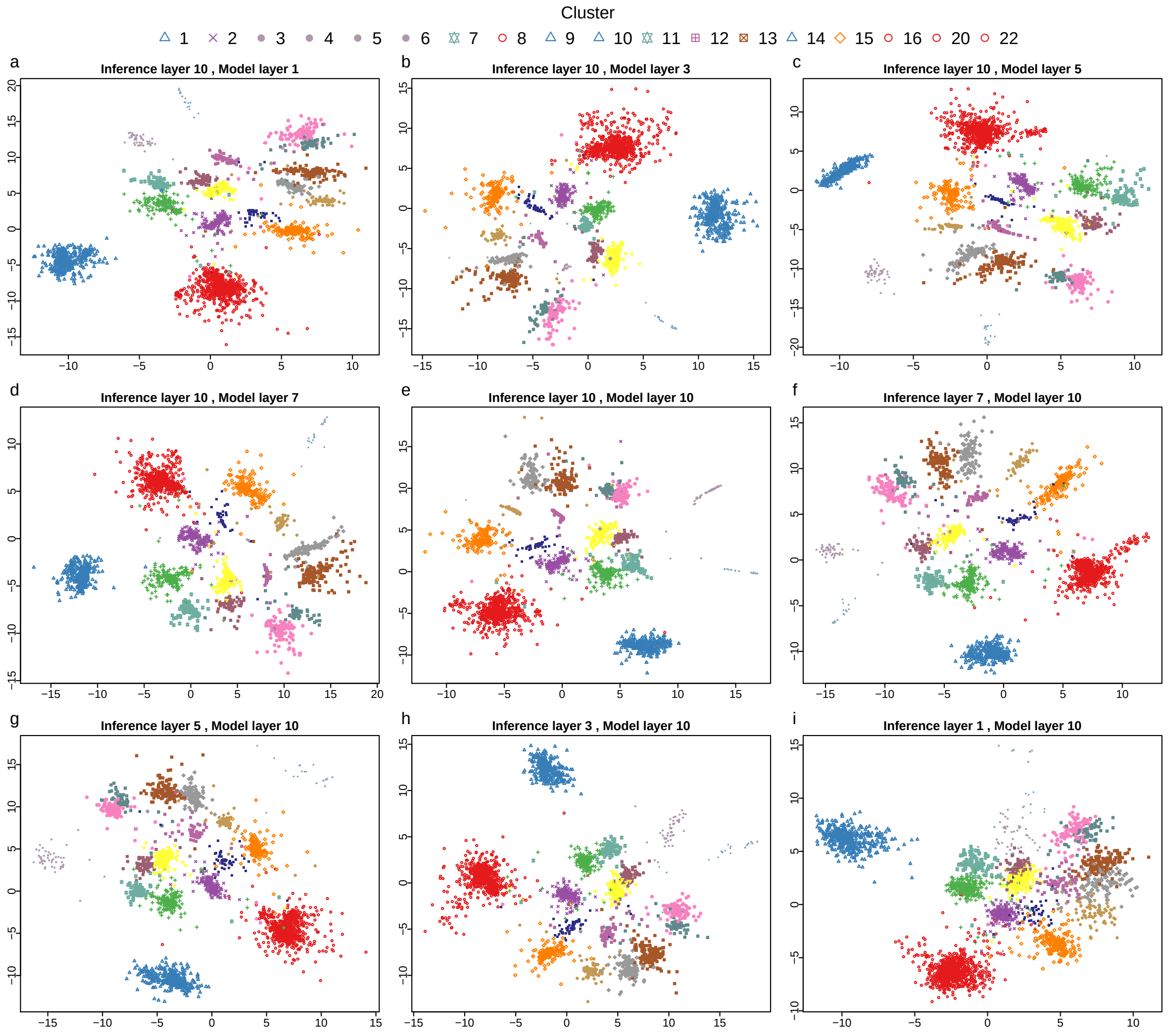

Supplementary Fig. 15: The influence of the number of layers in the neural networks on `scvis` results on held-out test data (by using the mapping functions from the training data). We used the held-out 3,111 test bipolar dataset from batch six. a-e) the inference networks have 10 layers, each layer has 128 units, and the model network has 1, 3, 5, 7, 10 layers, respectively. f-i) the model networks have 10 layers, each layer has 128 units, and the influence network has 7, 5, 3, 1 layers, respectively. Here the color and symbol combinations encode cells types (clusters).

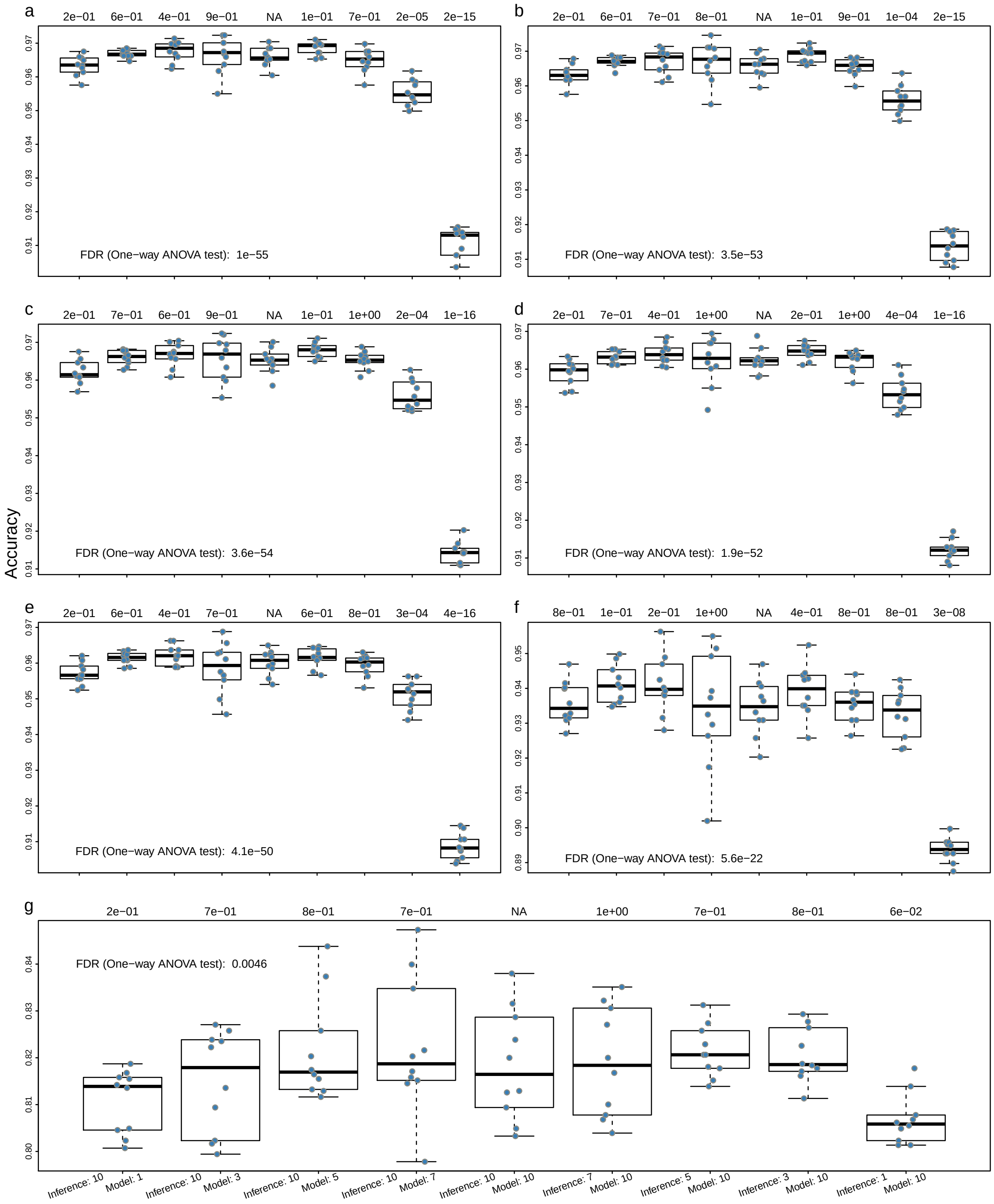

Supplementary Fig. 16: The influence of the number of layers in the neural networks on `scvis` results on held-out test data. The adjusted p-values (FDR, two-sided Welch's t-test, comparing the Knn classification accuracies from each model to those from the most complex model with both ten layers of model neural networks and variational influence neural networks) are shown on the top of each figure. We trained Knn classifiers on the two-dimensional representations of the subsampled data and tested on the held-out 3,111 bipolar dataset from batch six. a-g) the Knn classifier parameter K = 5, 9, 17, 33, 65, 129, and 257, respectively. Boxplots denote the medians and the interquartile ranges (IQR). The whiskers of a boxplot are the lowest datum still within 1.5 IQR of the lower quartile, and the highest datum still within 1.5 IQR of the upper quartile.

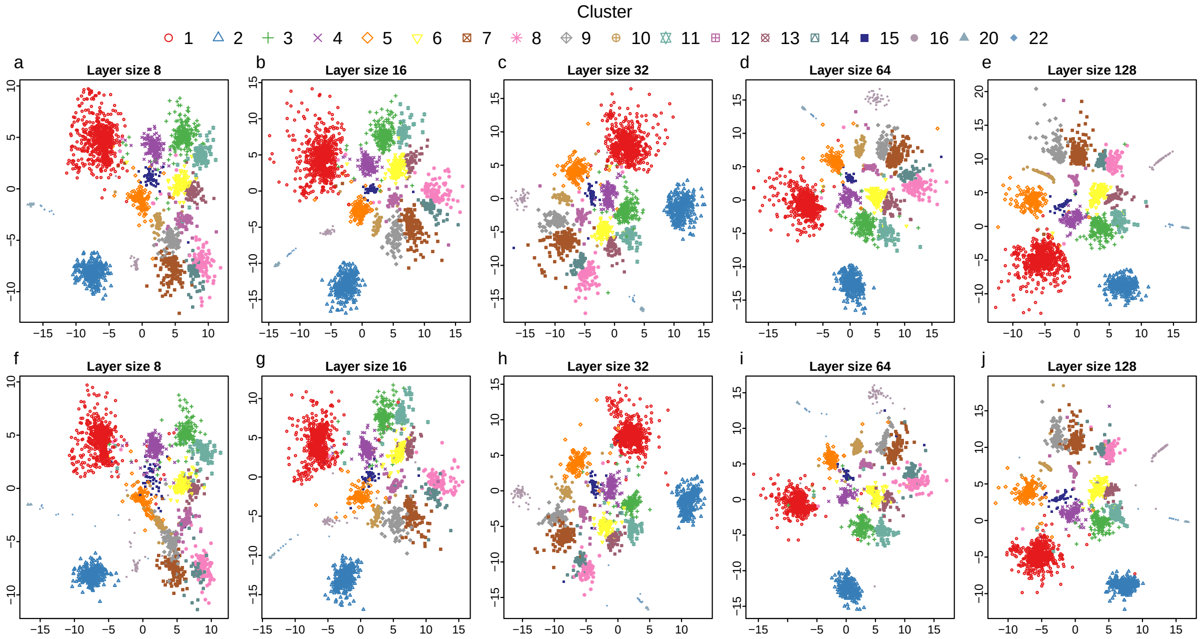

Supplementary Fig. 17: The influence of the layers sizes of the neural networks on `scvis` results. We used the subsampled 3,110 training bipolar dataset from batch six. For all experiments, both the model neural networks and the variational influence neural networks have ten layers. a-e) each layer of the neural networks have 8, 16, 32, 64, 128 units, respectively. f-i) The trained neural networks on the held-out out-of-sample data (by using the learned mapping function from the training data). Each layer of the neural networks have 8, 16, 32, 64, 128 units, respectively. Here the color and symbol combinations encode cells types (clusters).

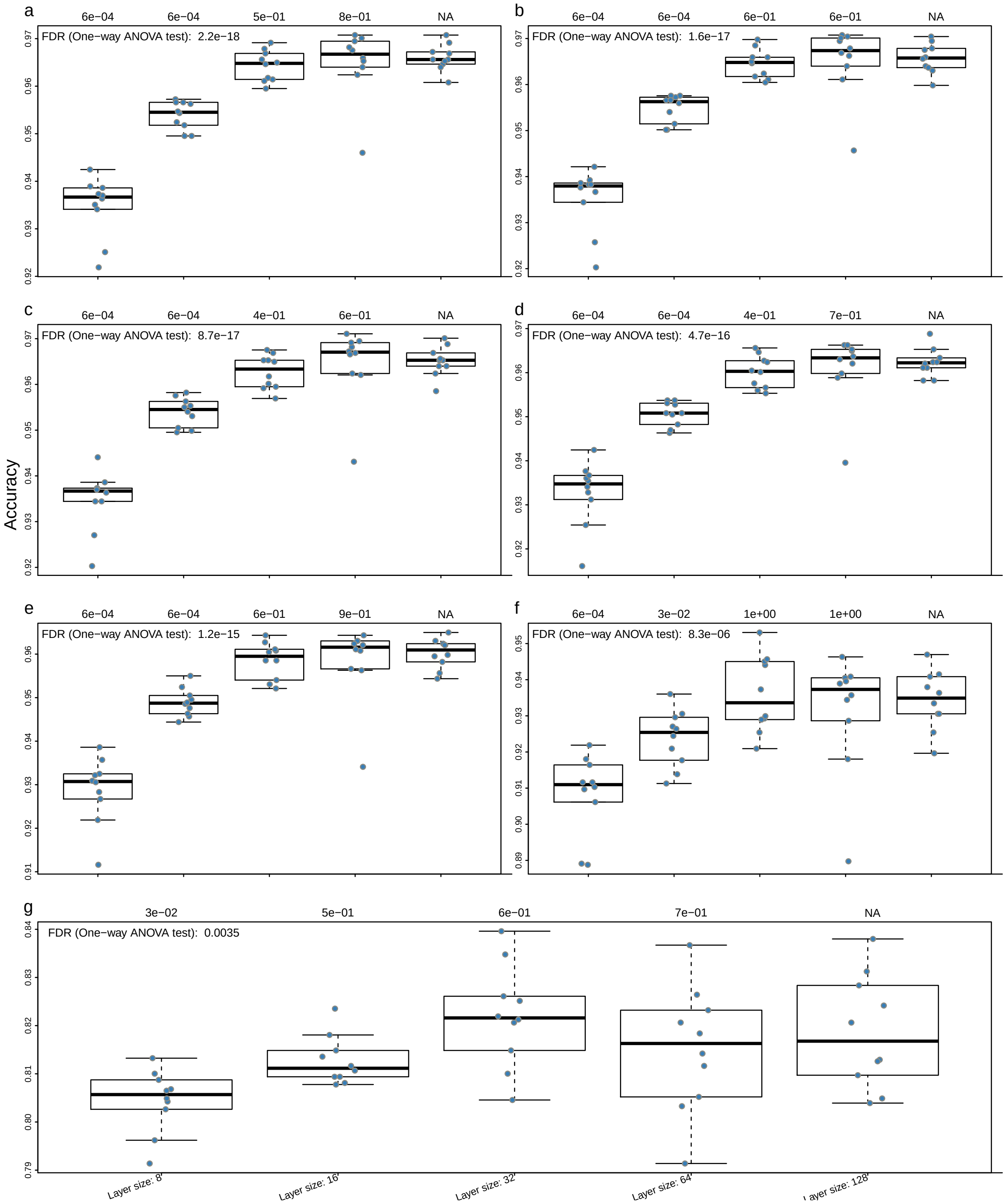

Supplementary Fig. 18: The influence of the layers sizes of the neural networks on `scvis` results. The adjusted p-values (FDR, two-sided Mann-Whitney U-test, comparing the Knn classification accuracies from each model to those from the most complex model with 128 units for each layer) are shown on the top of each figure. We trained Knn classifiers on the two-dimension embedding coordinates from the subsampled data and tested on the held-out 3,111 bipolar dataset (by using the mapping functions learned from the training data) from batch six. a-g) the Knn classifier parameter K = 5, 9, 17, 33, 65, 129, and 257, respectively. Boxplots denote the medians and the interquartile ranges (IQR). The whiskers of a boxplot are the lowest datum still within 1.5 IQR of the lower quartile, and the highest datum still within 1.5 IQR of the upper quartile.

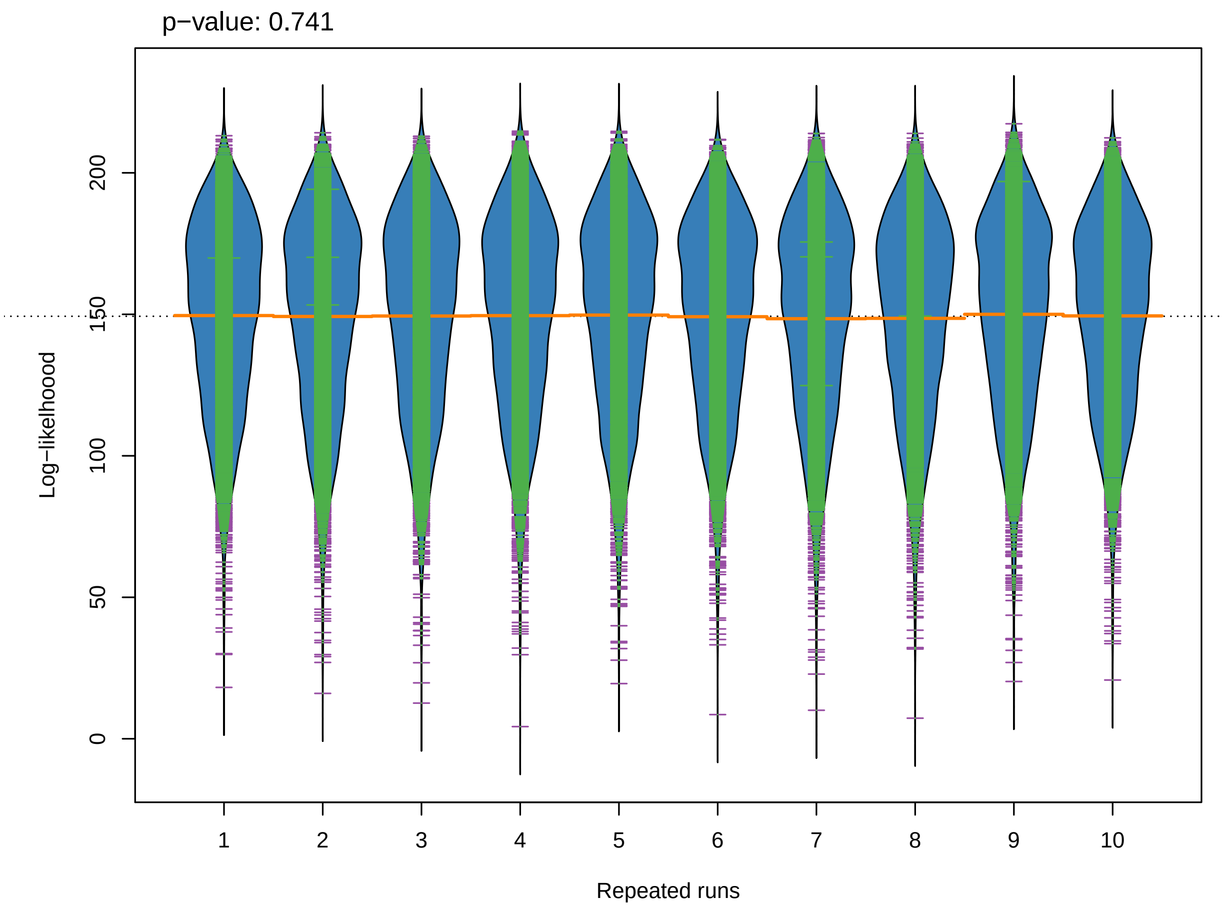

Supplementary Fig. 19: Beanplot shows the distributions of log-likelihoods obtained from ten repeated runs with layer size of 64. From the log-likelihoods, we cannot clearly see which one performs better. The one-way ANOVA test p-value is at the top left corner. The center orange lines of the beanplots denote the means.

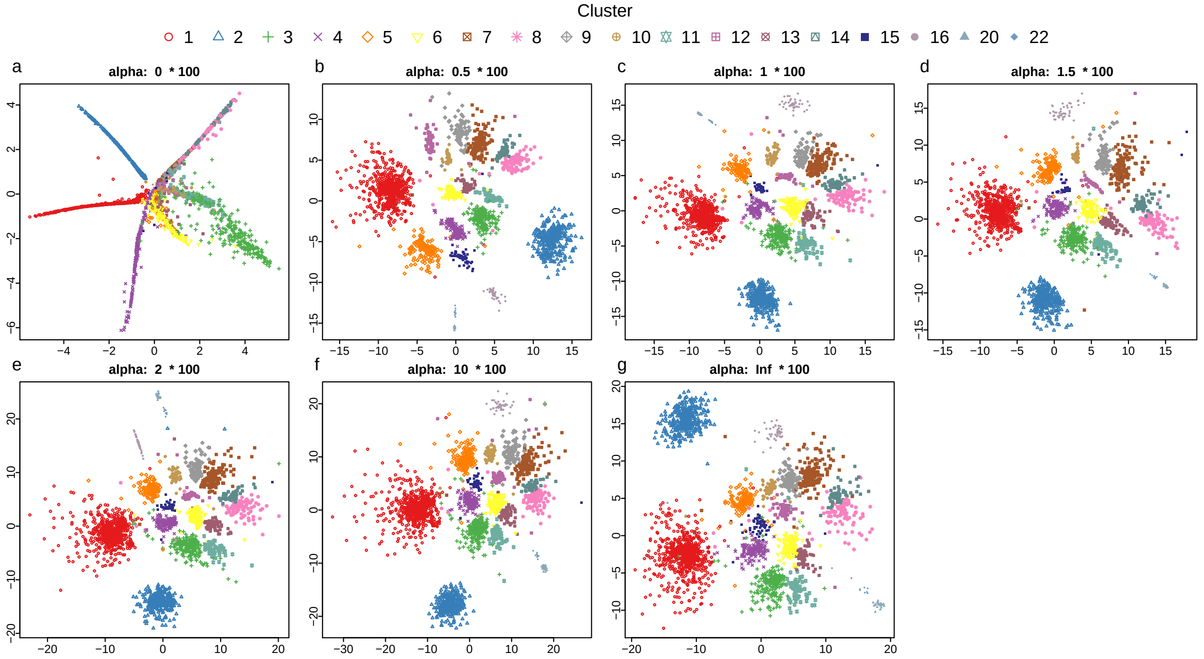

Supplementary Fig. 20: The influence of the α parameter on `scvis` results. We used the subsampled 3,110 training bipolar dataset from batch six. For all experiments, both the model neural networks and the variational influence neural networks have ten layers, and each layer has 64 units. a-g) the α parameter was set to 0, $0.5D$, $1D$, $1.5D$, $2D$, $10D$, and inf, where D is the dimensionality of the input data ($D = 100$ for the bipolar data by using the top 100 principal components). Here the color and symbol combinations encode cells types (clusters).

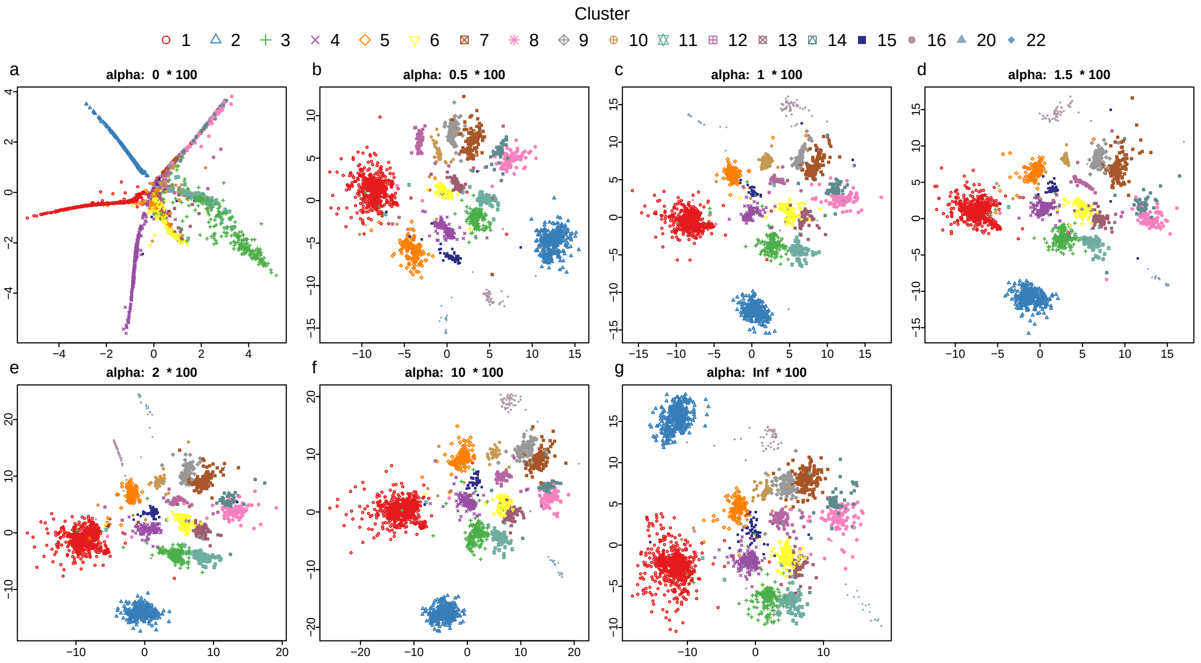

Supplementary Fig. 21: The influence of the α parameter on embedding the out-of-sample test data (by using the learned mapping functions). a-g) the α parameter was set to 0, $0.5D$, $1D$, $1.5D$, $2D$, $10D$, and inf, where D is the dimensionality of the input data ($D = 100$ for the bipolar data by using the top 100 principal components). Here the color and symbol combinations encode cells types.

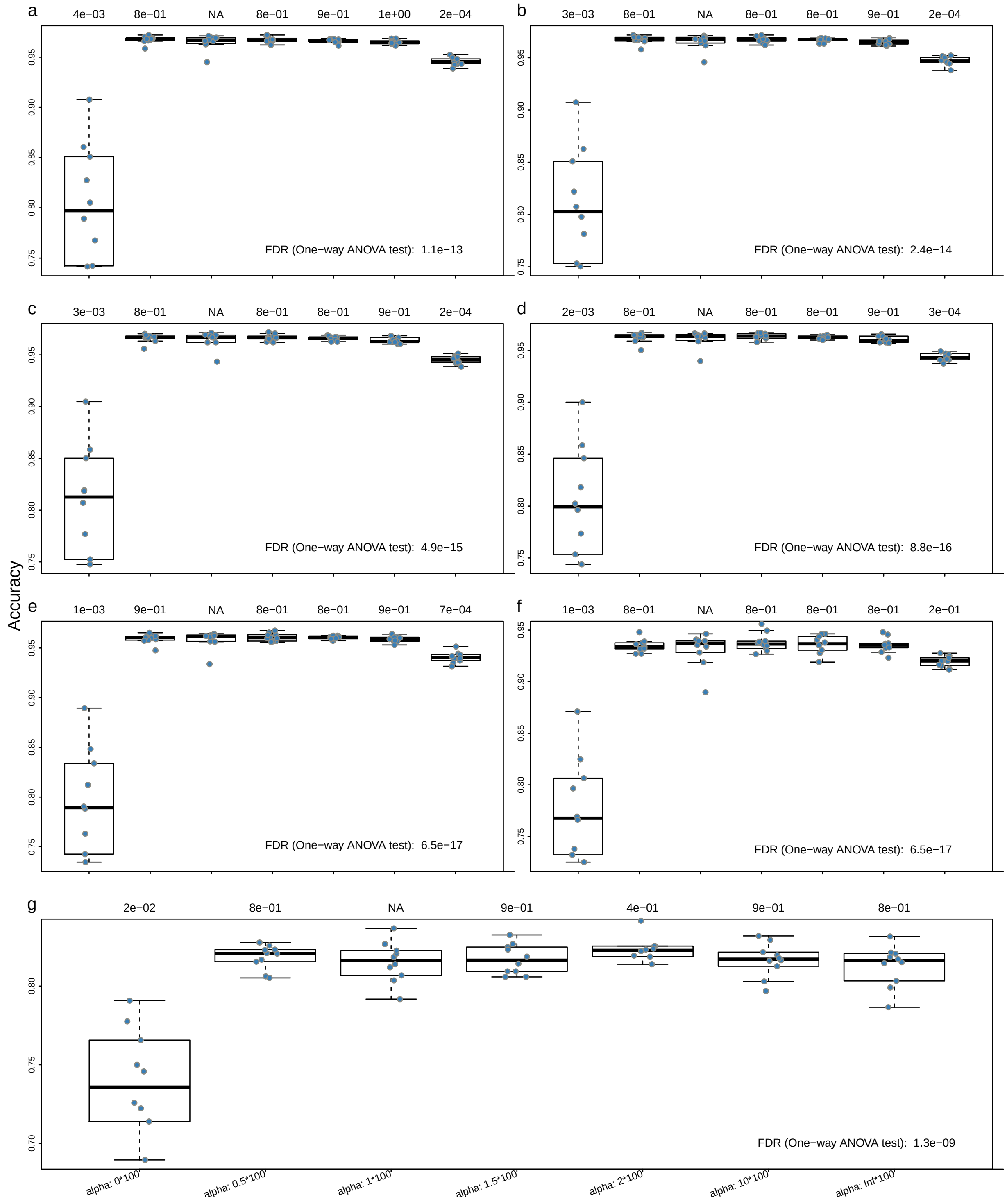

Supplementary Fig. 22: The influence of the α parameter on embedding the out-of-sample test data (by using the learned mapping functions). The adjusted p-values (FDR, two-sided Welch's t-test, comparing the Knn classification accuracies from each model to those from the default model by setting α to the dimensionality of the input data) were show on the top of each figure. We trained Knn classifiers on the two-dimension representations of the subsampled data and tested on the held-out 3,111 bipolar dataset from batch six. a-g) the Knn classifier parameter K = 5, 9, 17, 33, 65, 129, and 257, respectively. Boxplots denote the medians and the interquartile ranges (IQR). The whiskers of a boxplot are the lowest datum still within 1.5 IQR of the lower quartile, and the highest datum still within 1.5 IQR of the upper quartile.

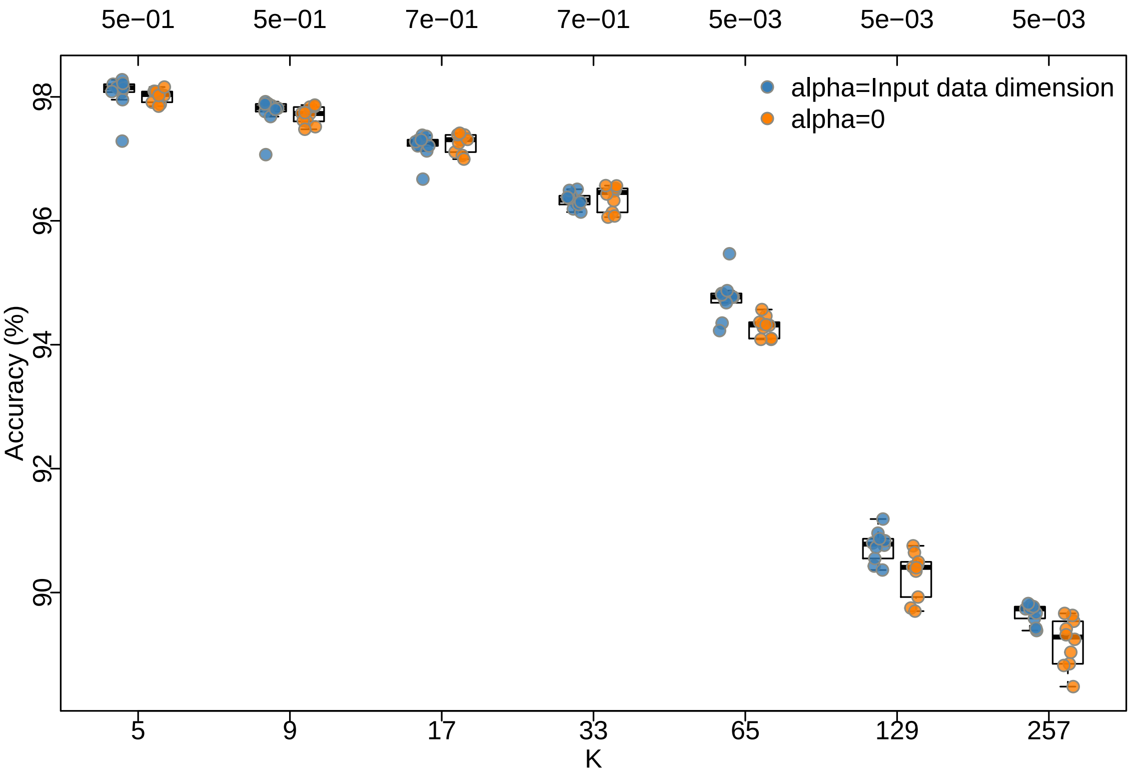

Supplementary Fig. 23: Comparing the influence of the α parameter on embedding new data using the nine-dimensional data. The adjusted p-values (FDR, one-sided Welch's t-test, comparing the Knn classification accuracies from each model to those from the default model by setting α to the dimensionality of the input data) are shown on the top of each figure. We trained Knn classifiers on the two-dimension embedding (the smaller synthetic dataset with 2,200 points) outputs from `scvis` and tested on the two-dimensional embedding of the larger 22,000 synthetic data. The Knn classifier parameter K = 5, 9, 17, 33, 65, 129, and 257. Boxplots denote the medians and the interquartile ranges (IQR). The whiskers of a boxplot are the lowest datum still within 1.5 IQR of the lower quartile, and the highest datum still within 1.5 IQR of the upper quartile.

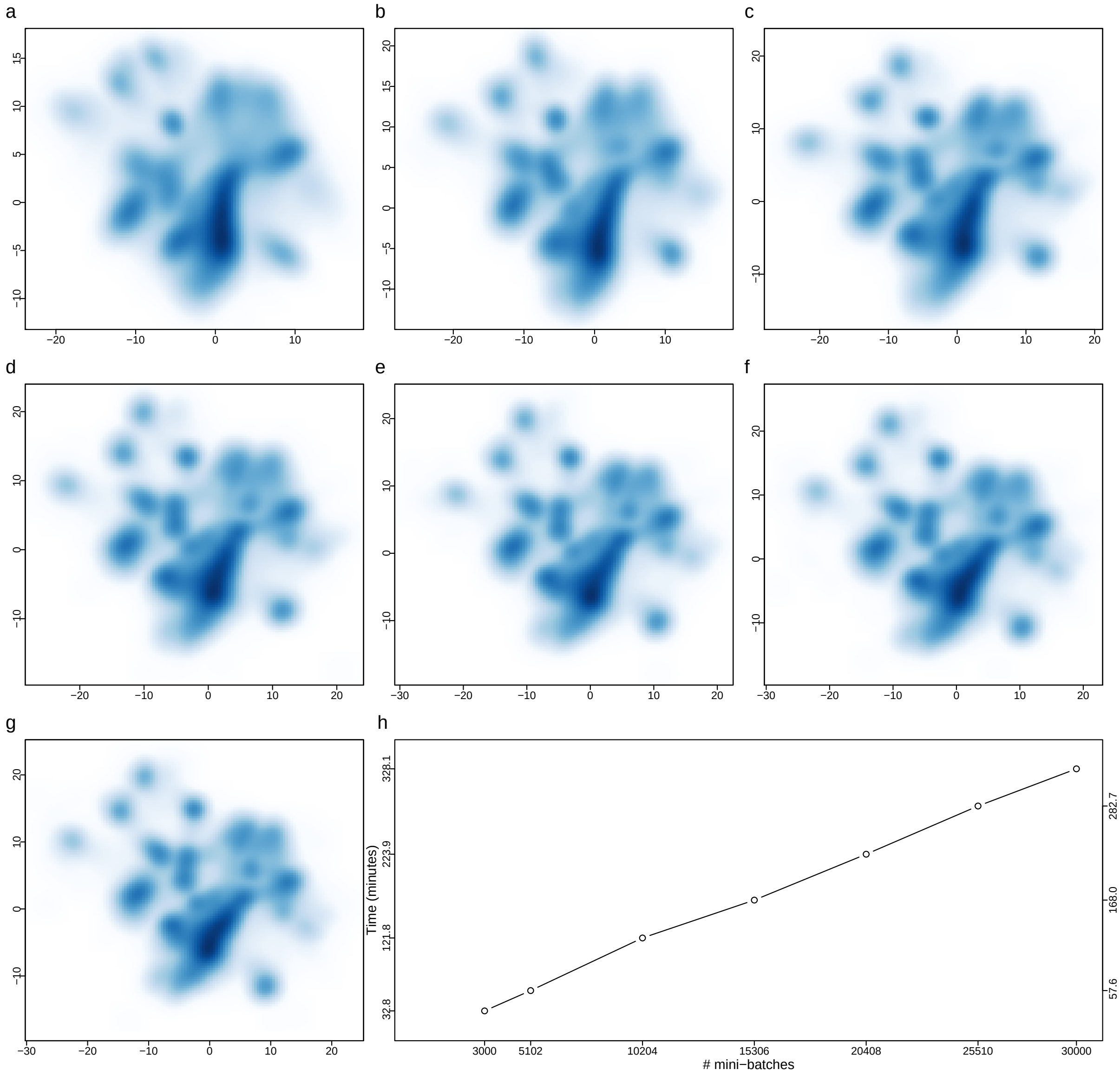

Supplementary Fig. 24: The running time and performance of `scvis` on the large 10X Genomics 1.3 million E18 neural cells. a -g) show the density of the `scvis` results at different number of training mini-batches, 3,000, 5,102, 10,204, 15,306, 20,408, 25,510, and 30,000 mini-batches. h) The time (in minutes) used to train `scvis` at a given number of mini-batches.
